# Dendritic Cell-Based Immunotherapy: The Importance of Dendritic Cell Migration

**DOI:** 10.1155/2024/7827246

**Published:** 2024-04-08

**Authors:** Min-Seon Song, Ji-Hee Nam, Kyung-Eun Noh, Dae-Seog Lim

**Affiliations:** Department of Bioconvergence, Graduate School and Department of Biotechnology, CHA University, 335 Pangyo-ro, Bundang-gu, Seongnam-si, Gyeonggi-do 13488, Republic of Korea

## Abstract

Dendritic cells (DCs) are specialized antigen-presenting cells that are crucial for maintaining self-tolerance, initiating immune responses against pathogens, and patrolling body compartments. Despite promising aspects, DC-based immunotherapy faces challenges that include limited availability, immune escape in tumors, immunosuppression in the tumor microenvironment, and the need for effective combination therapies. A further limitation in DC-based immunotherapy is the low population of migratory DC (around 5%–10%) that migrate to lymph nodes (LNs) through afferent lymphatics depending on the LN draining site. By increasing the population of migratory DCs, DC-based immunotherapy could enhance immunotherapeutic effects on target diseases. This paper reviews the importance of DC migration and current research progress in the context of DC-based immunotherapy.

## 1. Introduction

Dendritic cells (DCs) are immune cells that play a vital role in linking innate and adaptive immune responses to efficiently promote immune defense and maintain tolerance [1, 2]. DCs can be subdivided into distinct subsets, which include plasmacytoid DCs (pDCs) producing interferon (IFN), monocyte-derived DCs (Mo-DCs), classical or conventional DCs (cDCs), and Langerhans cells (LCs), which reside in the epidermis [3–5].

DCs internalize self and non-self antigens (Ags) and present them using major histocompatibility complex (MHC) molecules. Endogenous Ags are presented on MHC class I molecules to CD8^+^ T cells, whereas exogenous Ags are presented on MHC class II molecules to CD4^+^ T cells [6, 7]. Following exposure to pathogen-derived Ags, DCs undergo maturation, during which the secretion of inflammatory cytokines, the expression of costimulatory molecules, and MHC molecules increase. Several factors can affect the maturation of DCs; e.g., exposure to lipopolysaccharide (LPS) stimulates the Toll-like pattern recognition receptor (TLR)-4 to increase downstream signaling that activates the nuclear factor kappa B (NF*κ*B) transcription factor. Mature DCs (mDCs) upregulate chemokine receptors, such as C-C chemokine receptor type 7 (CCR7), and then migrate to draining lymph node (dLN), which is essential for initiating T-cell-mediated immune response [1, 6].

DC migration through lymphatic vessels toward secondary lymphoid organs involves changes in the expression of adhesion molecules and chemokine receptors and cytoskeleton reorganization. This enables the differentiation of immature DCs (imDCs) in peripheral tissues and the migration of Ag-transporting DCs to afferent lymphatics and Ag-presenting mDCs to lymphoid tissues [8].

DC migrates to dLN to encounter naïve T cells bearing Ag-specific T-cell receptors (TCRs). DCs present Ag to T cells through MHC molecules. These interactions between DC-T cells, the so-called immunological synapse (IS), require three sequential signals: TCR recognition of Ags (Signal 1), signal transduction between DC-T cells using costimulatory molecules (Signal 2), and cytokine release (Signal 3). A lack of costimulation can lead to anergy or tolerance of T cells [3, 9, 10]. This signal guides T cells to develop into Ag-specific effector types; CD4^+^ T cells differentiate into effector helper T (Th) cells, while CD8^+^ T cells differentiate into cytotoxic T lymphocytes (CTLs) [8, 11]. The DC network efficiently promotes T-cell-mediated immunity, allowing selective targeting and direct elimination of infected or cancerous cells. Additionally, activated T cells can migrate to nonlymphoid tissues, further contributing to their effector functions at various locations [8].

DC-based immunotherapy has significant potential for the treatment of cancer and other diseases. Although DC-based immunotherapies have shown promise, it is important to note that the field of immunotherapy is rapidly evolving, and the effectiveness of these approaches can vary depending on the type of disease, patient characteristics, and other factors [12, 13]. Improving DC migration to LN could enhance DC-mediated immune responses by providing an Ags to T cells. DC migration is a significant factor that contributes to activating the T-cell-mediated immune system, targeting not only cancer but also inflammation and other specific disorders. Therefore, this review focuses on the crucial role of DC migration in DC-based immunotherapy.

## 2. History of DC-Based Immunotherapy

### 2.1. DC-Based Anticancer Immunotherapy

Cancer treatments have continuously evolved and advanced. In the 1800s, surgery focused on removing tumors and nearby tissues to halt cancer cell progression and spread, although this did not limit the risk of cancer recurrence, particularly in metastatic cases. During the 1900s, X-rays or radioactive isotopes were used in radiation therapy to target cancer cells, but could also damage neighboring organs [14]. In the 2000s, targeted cancer therapy involved the disruption of specific pathways linked to carcinogenesis and tumor growth [15]. Since the 2010s, immunotherapeutic treatments have employed immunomodulatory approaches designed to enhance overall immune responsiveness, amplifying anticancer immune responses [16]. Therefore, novel therapeutic approaches are required to improve antitumor responses.

mDCs have been used for cancer immunotherapy and have demonstrated therapeutic potential against metastatic tumors. Subcutaneous vaccination with tumor lysate-pulsed DCs minimized or prevented the spread of pulmonary metastatic nodules and partially inhibited tumor growth in mice [17]. Additionally, cDC1 vaccination induces antitumor CD8^+^ CTLs, whereas cDC2 vaccination reduces tumor growth and promotes Th17 cells in mice [18]. DCs have also shown therapeutic effects in cancers, such as colorectal, pancreatic, and breast cancer [19–21], although DC-based immunotherapy has not achieved the expected efficacy in most solid tumors, even in murine models. However, coadministration of cytokines with DC vaccination has shown promising results, significantly suppressing tumor growth and improving in vivo immune responses [22]. In order to enhance the longevity of DCs in vivo and promote increased migration, one approach is to coadminister cytokines, such as inflammatory cytokines (e.g., interleukin (IL)-2, IL-12, and IFN), as adjuvants with a DC vaccination [22–25]. Thus, DC vaccination that enhances DC migration through coadministration with cytokines may become a novel approach in cancer immunotherapy.

### 2.2. DC-Based Treatment of Inflammatory and Autoimmune Diseases

Autoimmune diseases are pathological conditions that affect 3%–10% of the general population and are characterized by dysregulated inflammation against auto-Ags, where the immune system mistakenly attacks its own healthy cells and organs, leading to inflammation and damage [26]. Immunosuppressive drugs, such as azathioprine and methotrexate, were introduced in the 1950s and 1960s and used to suppress the overactive immune response in autoimmune diseases, providing additional treatment options in addition to corticosteroids [27]. In the 21st century, targeted therapies have contributed to a new era of treating autoimmune diseases. Biological drugs, such as tumor necrosis factor (TNF) inhibitors, IL blockers, and B-cell inhibitors, have gained widespread use, offering more precise and effective treatments with fewer side effects than traditional immunosuppressive drugs [28].

Conventional treatments for autoimmune diseases involve suppressing general immune function to modulate uncontrolled inflammation. Tolerogenic DCs (tDCs) are characterized by reduced expression of costimulatory molecules and secretion of proinflammatory cytokines, such as IL-12, while exhibiting elevated production of anti-inflammatory cytokines, such as IL-10 [11]. For example, CD103^+^ DC, one of the cDC2 subsets in the gut, can promote immune tolerance by inducing regulatory T (Treg) cells [3]. In the mouse spleen, the cholesterol regulatory pathway that involves liver X receptor (LXR) *α*/*β* also can control the immunogenic and tolerogenic cDC maturation [29]. These tDCs are instrumental in maintaining immune homeostasis by activating Treg cells, inhibiting effector T cells, and modulating Th1/Th2 immune responses [30, 31]. tDCs play an important role because they are used as anti-inflammatory and immunosuppressive targets in several models of autoimmune diseases, contributing to the induction of peripheral tolerance [26, 30].

Additionally, tDC-based therapy is applicable to autoimmune diseases, including rheumatoid arthritis (RA), systemic lupus erythematosus, Crohn's disease, and type 1 diabetes mellitus (T1DM) [31–35]. Monocyte-derived tDCs (Mo-tDCs) from patients with RA exhibit a tolerogenic phenotype characterized by reduced levels of costimulatory molecules, low production of proinflammatory cytokines, and impaired stimulation of autologous Ag-specific T cells comparable with that of healthy controls. Furthermore, tDCs suppress mDC-induced T-cell proliferation and IFN-*γ* and IL-17 production [33]. The therapeutic potential of tDCs has been demonstrated in experimental autoimmune myocarditis by inducing Treg cells belonging to the CD4^+^CD25^+^ subset [30]. Moreover, in acute myocardial infarctions (AMIs), heart tissue-specific Mo-tDCs generate Treg cells that can be used for effective therapeutic remodeling after MI [36]. For autoimmune diseases, increasing the migration of tDC may produce more effective treatment outcomes than those currently observed.

### 2.3. DC-Based Immunotherapy: Features and Limitations

DC-based immunotherapy is a cutting-edge approach to disease treatment that harnesses the power of the patient's own immune system. The process involves isolating DCs from the patient's blood or tissue and then modifying them in the laboratory to present tumor-specific Ags. These Ag-loaded DCs are reintroduced into the patient, effectively training the immune system to recognize and target cancer cells. Notably, an advantage of this personalized treatment is the induction of a targeted and robust immune response while minimizing harm to healthy cells [37]. Furthermore, the potential to generate memory T cells provides the opportunity for long-lasting protection against cancer recurrence. Although challenges remain, DC-based immunotherapy holds considerable promise in revolutionizing cancer care and offering new opportunities to patients [38].

However, DC-based immunotherapy is limited by the complex and labor-intensive process involved in generating personalized vaccines for each patient because specialized facilities and skilled personnel are required to harvest, manipulate, and load DC with Ags [12, 37, 38]. The personalized nature of treatment can be time-consuming and expensive, potentially delaying its administration to patients with rapidly progressing cancers. Furthermore, while DCs are crucial in activating T cells and initiating an immune response, cancer cells may evade the immune system [12, 39, 40].

Another limitation of DC-based immunotherapy is associated with DC migration. After administration, DCs must migrate efficiently to LNs to activate T cells and initiate a strong immune response. However, administered cells may not reach the LNs, causing potential inefficiencies in the immune activation process. Moreover, the tumor microenvironment can affect the effective presentation of Ags by affecting DC movement and function [41–44]. Ongoing research optimizes DC-based immunotherapy and overcomes these limitations to provide more effective and accessible treatment options. Current research is focused on developing strategies to improve DC migration and to optimize their therapeutic potential in the fight against cancer and other target diseases. Resolving these migration challenges is crucial to enhancing the overall efficacy of DC-based immunotherapy.

## 3. Migration of DCs

### 3.1. DC Maturation and Migration Processes

imDCs have a star-like morphology (so-called dendrites) with dendritic extensions. These extensions enable environmental surveying and perform broad movements to capture Ags within tissues or organs through processes such as phagocytosis or endocytosis. Consequently, imDCs need to modify their cytoskeleton to facilitate Ag engulfment [45].

In contrast, as DCs mature, their phagocytic activity decreases, whereas their mobility increases, and they exit from resident tissues. mDCs then migrate through lymphatic vessels to interact with T cells and trigger an adaptive immune response. DCs undergo substantial cytoskeletal rearrangements, primarily involving the actin cytoskeleton. This cytoskeletal reorganization plays a vital role in DC migration, Ag-presentation, cell–cell interactions, and IS formation, thus initiating T-cell priming [46–48]. Cortical stiffness in DCs is linked to the reorganization of their cytoskeleton. mDCs exhibit a higher level of cortical stiffness than imDCs [45, 49].

Migration of DCs from the injury site to the lymphatic vessel is dependent on the expression of CCR7, which is upregulated in mDCs in response to stimuli activating the DCs. In particular, CCR7 is crucial not only for the entry of DCs into the LN but also to facilitate the entry of naïve T cells into the same LN. CCR7 also facilitates the migration of DCs from the tumor microenvironment to the tumor-dLN [50, 51]. Therefore, the regulation of expression of CCR7 ultimately affects DC chemotaxis [52].

### 3.2. Influence of DC Migration on T Cells

DCs have limited ability to migrate to distant LNs but excel in migration through lymphatic vessels to reach nearby LNs [36], where they are particularly efficient in inducing Ag and/or tissue-specific T cells [45, 53]. The interaction between DCs and T cells, including the formation of an IS, is based on several factors, including the mechanical stiffness of DCs and the expression of costimulatory molecules, such as CD40 and CD80/CD86. Reducing the stiffness of DCs decreases their cytoskeleton dynamics, affects DC-T-cell interactions, and may result in a decline in the capacity to activate T cells. Costimulatory molecules expressed in DCs also play a pivotal role in the interaction between DCs and T cells [9, 10].

### 3.3. The Migratory Function of DCs and How to Increase Migratory Capacity

DC migration can be affected by various factors, such as cytokines and metabolites, which can decrease or increase DC motility [49, 54]. For example, nucleotide adenosine-5′-monophosphate (AMP) promotes the migration of humans imDC by triggering actin polymerization [55]. In addition, LCs, one of the specific subsets of DCs that reside in the epidermis, undergo maturation under the influence of well-known DC maturation-inducing cytokines, including IL-1*β* and TNF [56]. When a sufficient concentration of these cytokines is present in the intracellular environment, the activated DCs can be sustained for a prolonged period [52, 56].

In contrast, IFN-*β* inhibits bone marrow-derived DC migration in vitro and in vivo through signal transducer and activator of transcription (STAT)-1 signaling [57]. Prostaglandin D2 (PGD_2_) also impedes lung DC migration to the dLN [58]. A decrease in DC migration results in inadequate activation of Ag-specific effector T cells, reduces the number of effector T cells, and weakens the immune response. Most injected DCs die at the injection site, resulting in only approximately 10% of migratory DCs reaching the dLN, which is insufficient to induce differentiation of naïve T cells. Therefore, improving DC migration capacity can overcome these issues [41, 44].

### 3.4. Upregulation of F-Actin Rearrangement-Related Genes

During maturation, DCs undergo significant changes in their cytoskeletal organization. The actin cytoskeleton is critical for supporting cellular functions by controlling the structural integrity and movement of the cell and the localization, clustering, and stability of transmembrane proteins [46, 59, 60]. Actin-related protein 2/3 (Arp2/3) is a protein complex that assembles actin dimers, which regulate the actin cytoskeleton [61, 62]. Upregulating genes involved in F-actin, such as Arp2/3, to promote F-actin rearrangement could improve cytoplasmic extension of DC, potentially enhancing DC migration.

### 3.5. Cytokine-Induced Upregulation of Adhesion Molecules

Inflammation triggers the release of inflammatory cytokines, such as IL-1*β* and IL-12, by immune cells, allowing the activation of lymphatic endothelial cells [63]. This activation prompts increased adhesion molecule expression, such as vascular cell adhesion molecule 1 (VCAM-1) and intracellular adhesion molecule 1 (ICAM-1) [63, 64]. Integrins, which are expressed on DCs, are adhesion molecules that play a crucial role in DC migration. Lymphocyte function-associated Ag 1 (LFA-1; CD11a, *α*L*β*2 integrin) mediates DC binding to ICAM-1 on the surface of lymphatic endothelial cells, facilitating DC migration into lymphatic vessels and toward LN. Very late Ag-4 (VLA-4; CD49d/CD29, *α*4*β*1) is also involved in adhesion of DC to VCAM-1 expressed on lymphatic endothelial cells, promoting DC migration [64, 65]. These integrins facilitate the attachment of DCs to endothelial cells, allowing their migration from peripheral tissues to LNs, where they play essential roles in immune surveillance and activation [64–66]. Consequently, this establishes durable intracellular adhesion, promoting improved transmigration of DCs and facilitating their efficient transit to the dLN through interactions with integrin ligands on DCs.

### 3.6. Appropriate Expression of Chemokine Receptors

Chemokine receptors are G protein-coupled receptors (GPCRs) with seven-transmembrane-spanning domains. Chemokine receptor-expressing DCs migrate to dLN to bind to chemokines [63, 67–70]. Upon exposure to the C–C motif chemokine ligand (CCL)-19 of the CCR7 ligand, the DCs undergo internalization of both the receptor and the ligand. Through this internalization process, DCs generate a self-generated chemokine gradient. However, when chemokine gradients are low, DCs are difficult to perceive, whereas high gradients lead to receptor saturation, hindering cells from sensing spatial differences [71–73]. Stable gradients of CCL19 are crucial for stabilizing directionality because disruption of these gradients leads to impaired cytoskeletal polarization and persistent chemotaxis. Therefore, it may be important for DC migration that DCs properly express CCR7 to enable the formation of self-generated chemokine gradients.

### 3.7. Migration-Related Factors in DC

DC migration can be regulated through various factors such as chemokine receptors, genes, and cytokines that are involved in DC migration.

CCR7 can trigger DC migration from the epidermis and dermis to CCL-21-expressing lymphatic vessels within the dermis [74]. Additionally, CCR7 can also induce the migration of small intestinal DC to mesenteric LNs [67]. In cancer, CCL25/CCR9 coordinates the recruitment of pDCs to metastatic LN and carcinoma tissues [75]. The migration of pDCs is also influenced by type I IFN (IFN-I) [76]. Furthermore, IL-10 and tumor growth factor (TGF)-*β*1 promote cellular adhesion while inhibiting DC migration to dLN [77]. Ectonucleotide pyrophosphatase/phosphodiesterase 2 (Enpp2) facilitates the conversion of lysophosphatidylcholine (LPC) into lysophosphatidic acid (LPA), thus enhancing the migratory activity of Mo-DCs [78]. PDZ and Lim domain protein 4 (Pdlim4) governs F-actin rearrangement and is involved in dendrite formation and is involved in CCR-JNK-mediated migration of Mo-DC [46]. Prostaglandin E2 (PGE_2_) stimulates CCR7 expression in Mo-DC, causing a reorganization of the F-actin cytoskeleton and regulation of DC migration [60, 79]. Therefore, regulation of the factors involved in DC migration could potentially increase the capacity for DC migration.

## 4. Clinical Trials DC-Based Immunotherapy

### 4.1. Clinical Trials of DC-Based Anticancer Immunotherapy

**Figure 1:**
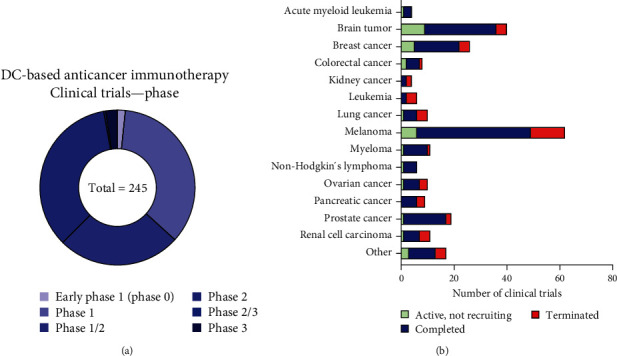
Clinical trials of DC-based antitumor immunotherapies: (a) DC-based anticancer immunotherapy was reported in different stages in clinical trials until July 2023; (b) distribution of status in current clinical trials among different types of cancer. “Other” includes bladder cancer, Ewing's sarcoma, gastric cancer, head and neck cancer, hepatocellular carcinoma, liver cancer, malignant mesothelioma, soft tissue sarcoma, and uveal melanoma. DC; dendritic cell.

Among the total of 245 clinical trials currently underway that use immunotherapy to target cancer (as of July 2023), four trials are in early phase 1 (2%), 86 trials are in phase 1 (35%), and 63 trials are in phase 1/2 (26%). Furthermore, 85 trials are in phase 2 (35%), one trial is in phase 2/3, and six trials are in phase 3 (2%). Phases 1 and 2 account for approximately 96% of these current trials (Figure 1(a)). Currently, clinical trials using DC-based approaches are underway for various types of cancer, and a significant number of trials are ongoing for melanoma, brain tumors, breast cancer, and prostate cancer, in that order. Furthermore, the status of trials based on cancer types shows that most of the trials have been completed (Figure 1(b)). Moreover, to improve the efficacy of cancer treatment, DCs are coadministered with current anticancer drugs or concomitantly administered with cytokines such as IFN-I as adjuvants [22–25, 76, 80–82]. However, there are no clinical results using adjuvants to improve DC migration.

### 4.2. Clinical Trials of DC-Based Autoimmune and Inflammatory Diseases

Thirteen clinical trials were found to be ongoing for targeting autoimmune diseases using DC-based approaches (Table 1). Five clinical trials target multiple sclerosis, with one study of unknown status, one study not yet recruiting, two studies currently recruiting, and one study completed. For neuromyelitis optica, one clinical trial has been completed. Three clinical trials are focused on RA, with one study of unknown status, one study currently recruiting, and one study completed. For T1DM, five clinical trials are reported, with two studies of unknown status, one study not yet recruiting, one study currently recruiting, and one study completed. Most of the clinical phases represented are in Phase 1 or 2. In clinical trials targeting autoimmune diseases, clinical progression has been conducted using tDC, which secretes anti-inflammatory cytokines (e.g., IL-10 and TGF-*β*) and induces immune tolerance. In ongoing clinical trials, the majority involve the administration of tDC alone. In research targeting autoimmune diseases, there are no clinical results that focus on adjuvants to enhance DC migration.

## 5. Conclusions and Future Perspectives

**Figure 2:**
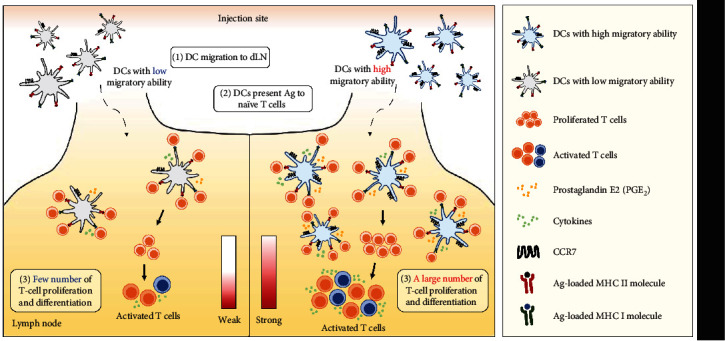
Increased DC migration induces more effective therapeutic effects. After internalization of the antigen (Ag), DCs mature and migrate to naïve T cells in the draining lymph node (dLN) to present Ag. DCs with enhanced migratory ability moving to LNs more extensively increase the proliferation and differentiation. Conversely, DCs with low migratory ability produce less DC migration to the LN, making it difficult to promote T-cell proliferation.

DC-based immunotherapy is influenced by the migratory function of DCs. After administration, most DCs lose viability at the injection site, and only around 5%–10% of DCs migrate to the LNs [41, 44]. Therefore, increasing the population of migratory DCs to more than 10% has potential as an alternative to overcome the current limitations of DC-based immunotherapy. However, a clinical trial administration of IFN-*α* (one of IFN-I) together with DCs, no clinical studies focusing on the migratory function of DC are currently ongoing [22]. Regulating the migratory capacity of DCs by expressing the factors involved in DC migration (Table 2) or enhancing the migration capacity of DCs in clinical settings could promote a more effective DC-based therapeutic outcome. In conclusion, we suggest that increasing the migratory capacity of DCs may enhance therapeutic effects not only for cancer but also for other target diseases (Figure 2).

## Figures and Tables

**Table 1:** Clinical trials of DC-based autoimmune diseases treatments.

Conditions	NCT number	Study title	Interventions	Phases	Study status	Enrollment	Study results
Multiple sclerosis	NCT02618902	A “Negative” dendritic cell-based vaccine for the treatment of multiple sclerosis: a first-in-human clinical trial	Tolerogenic dendritic cells (tolDCs)	Phase 1	Unknown	9	No
	NCT05451069	Neurofilament light chain, chitinase-3 like-1 proteins, and plasmacytoid dendritic cells in multiple sclerosis	Neurofilament light chain, chitinase-3 like-1 protein levels, tolerogenic plasmacytoid dendritic cells	N/A	Not yet recruiting	42	No
	NCT02903537	Tolerogenic dendritic cells as a therapeutic strategy for the treatment of multiple sclerosis patients (TOLERVIT-MS)	Autologous VitD3 tolerogenic monocyte-derived dendritic cells loaded with a pool of myelin peptides (tolDC-VitD3), interferon (IFN)-*β*	Phase 1	Recruiting	16	No
	NCT04530318	Dendritic cell therapy combined with immunomodulatory treatment in multiple sclerosis	Autologous peripheral blood differentiated adult dendritic cells expanded	Phase 2	Recruiting	45	No

Multiple sclerosis, neuromyelitis optica	NCT02283671	Treatment of multiple sclerosis and neuromyelitis optica with regulatory dendritic cell: clinical trial phase 1B	tolDCs loaded with myelin peptides	Phase 1	Completed	20	No

Rheumatoid arthritis	NCT03337165	Autologous tolerogenic dendritic cells for treatment of patients with rheumatoid arthritis	tolDCs	Phase 1	Completed	10	No
	NCT01352858	Autologous tolerogenic dendritic cells for rheumatoid arthritis (AutoDECRA)	tolDCs, arthroscopy, saline irrigation alone	Phase 1	Unknown	15	No
	NCT05251870	Tolerogenic dendritic cell therapy for rheumatoid arthritis	Autologous mature tolerogenic monocyte-derived dendritic cells loaded with the B29 peptide of HSP70	Phase 1/ phase 2	Recruiting	18	No

Type 1 diabetes mellitus	NCT05207995	The treatment of patients with type 1 diabetes mellitus with autologous tolerogenic dendritic cells	Autologous tolerogenic dendritic cells	Phase 1/ phase 2	Not yet recruiting	30	No
	NCT01947569	Dendritic cells for type 1 diabetes mellitus (T1DM) therapy	Immunoregulatory dendritic cells (iDCs)	Phase 1/ phase 2	Unknown	90	No
	NCT00445913	Autologous dendritic cell therapy for type 1 diabetes suppression: a safety study	Diabetes-suppressive dendritic cell vaccine	Phase 1	Completed	10	No
	NCT02354911	Autologous immunoregulatory dendritic cells for type 1 diabetes therapy	iDCs	Phase 2	Unknown	24	No
	NCT04590872	An immunotherapy vaccine (PIpepTolDC) for the treatment of patients with type 1 diabetes	tolDCs vaccine	Phase 1	Recruiting	7	No

**Table 2:** DC migration-related factors.

Classification	Name	Characteristics	References
Chemokines/cytokines	CCR7	(i) Induction of DC migration from the skin to the LN via CCL21-expressing lymphatics (ii) Lamina propria (LP)-DC in the small intestine induces migration of mDC toward CCL21	[67, 74]
	CCR8	(i) Promotion of CD301b^+^ cDC2 migration	[83]
	CCL25/CCR9	(i) Plasmacytoid DCs (pDCs) recruited in metastatic LNs and carcinoma tissue	[75]
	IFN-I	(i) Induction pDC migration	[76]
	IL-10	(i) Through enhancing adhesion, impairment the migration ability of mDCs	[77]
	TGF-*β*1	(i) Inhibition of DC migration from tumors into tumor-dLN	[84]
	TNF	(i) Induction LCs migration to dLN	[85]

Genes/proteins	Enpp2	(i) Increase of mDC migration activity	[78]
	Fascin1	(i) Podosome disassembly in mDC and cDC1	[86, 87]
	Pdlim4	(i) Involvement of CCR-JNK-mediated migration of mDCs (ii) Regulation of F-actin rearrangement and dendrite formation	[46]
	PD-L1	(i) Loss of PD-L1, impairs dermal DC migration from skin to LN	[88]
	*β*2 integrin	(i) CD11c^+^CD103^+^ DC recruitment to the infection site by *Trichuris muris*	[64]

Receptors	Met	(i) Enhancement of DC adhesion to laminin (ii) Induction of emigration of DC/LC from the epidermis	[89–91]
	PTPN12	(i) Induction of DC migration to dLN (ii) Augmentation of phosphorylation of PYK2 and paxillin	[92]

Other	PGE_2_	(i) Induction of CCR7 expression in monocyte-derived DCs (MoDCs) (ii) Reorganizing the F-actin cytoskeleton, regulating the migration of DCs	[60, 79]
	lnc-DPf3	(i) CCR7-induced DC migration (ii) Suppression DC migration by inhibiting HIR-1*α*-dependent glycolysis	[68]

*Abbreviations*. CCR7, C–C chemokine receptor type 7; CCL25, C–C motif chemokine ligand 25; IFN-I, type 1 interferon, IL-10, interleukin 10; TGF-*β*1, tumor growth factor *β*1; TNF, tumor necrosis factor, Enpp2, ectonucleotide pyrophosphatase/phosphodiesterase 2; Fascin1, Fascin actin-bundling protein 1; Pdlim4, PDZ and Lim domain protein 4; PD-L1, programmed death-ligand 1; Met, mesenchymal epithelial transition; PTPN12, protein tyrosine phosphatase nonreceptor type 12; PGE_2_, prostaglandin E2; lnc-DPf3, long noncoding RNA DPf3; LP-DC, lamina propria-DC; pDCs, plasmacytoid DCs; LCs, langerhans cells; dLN, draining LN; MoDCs, monocyte-derived DCs.

## Data Availability

No data are used or generated during this study.

## Abbreviations

DCs: Dendritic cells
IFN: Interferon
pDCs: Plasmacytoid DCs
MoDCs: Monocyte-derived DCs
cDCs: Conventional DCs
LCs: Langerhans cells
Ags: Antigens
MHC: Major histocompatibility complex
LPS: Lipopolysaccharide
TLR: Toll-like pattern recognition receptor
NF*κ*B: Nuclear factor kappa B
mDCs: Mature DCs
CCR: C–C chemokine receptor
dLN: Draining lymph node
imDCs: Immature DCs
TCRs: T-cell receptors
IS: Immunological synapse
Th cells: Helper T cells
CTLs: Cytotoxic T lymphocytes
NK cells: Natural killer cells
IL: Interleukin
TNF: Tumor necrosis factor
tDCs: Tolerogenic DCs
Treg cells: Regulatory T cells
LXR: Liver X receptor
RA: Rheumatoid arthritis
T1DM: Type 1 diabetes mellitus
Mo-tDCs: Monocyte-derived tDCs
AMIs: Acute myocardial infarctions
AMP: Nucleotide adenosine-5′-monophosphate
STAT: Signal transducer and activator of transcription
PGD_2_: Prostaglandin D2
Arp2/3: Actin-related protein 2/3
VCAM-1: Vascular cell adhesion molecule 1
ICAM-1: Intracellular adhesion molecule 1
LFA-1: Lymphocyte function-associated antigen 1
VLA-4: Very late antigen-4
GPCRs: G protein-coupled receptors
CC: C–C motif chemokine ligand
TGF: Tumor growth factor
Enpp2: Ectonucleotide pyrophosphatase/phosphodiesterase 2
LPC: Lysophosphatidylcholine
LPA: Lysophosphatidic acid
Pdlim4: PDZ and Lim domain protein 4
PGE_2_: Prostaglandin E2.
